# Supplementary material for: Genomic diversity in Paenibacillus polymyxa: unveiling distinct species groups and functional variability
Source: BMC Genomics. 2024 Jul 25;25:720. doi: 10.1186/s12864-024-10610-w (PMC11271205; doi:10.1186/s12864-024-10610-w)
Supplement: Supplementary file 1 — Supplementary Material 1 [file 12864_2024_10610_MOESM1_ESM.docx]

# Supplemental figures


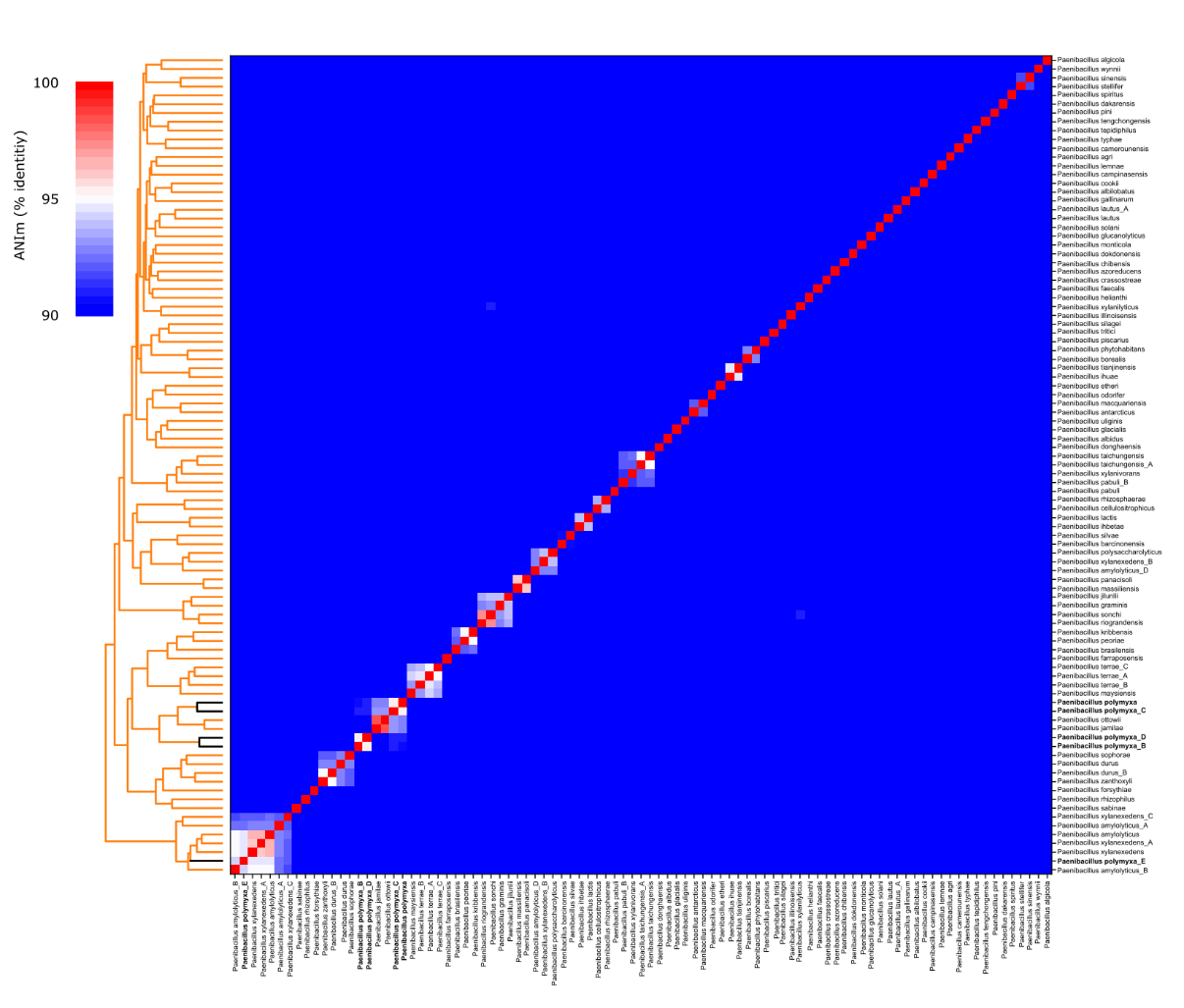


**Figure S1 Average nucleotide identity results for 93 Paenibacillus species representative strains based on mummer comparisons**. The representative strains of GTDB-validated Paenibacillus species were selected for this analysis. Strains of the different P. polymyxa groups are indicated in bold and black bars on the dendrogram.


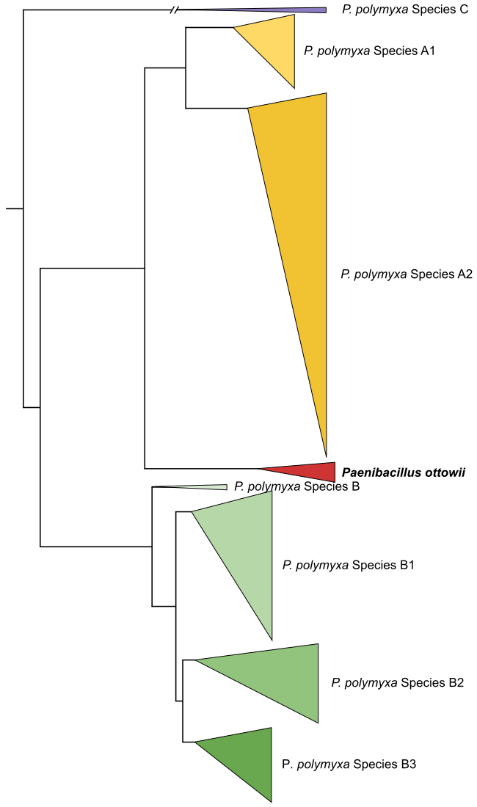


**Figure S2 Phylogenetic reconstruction P. polymyxa species groups and P. ottowii.** Single copy orthologous protein sequences were detected and aligned for 157 P. polymyxa genomes and 5 P. ottowii genomes using Orthofinder resulting in an alignment of 1388 sequences for a total of 403,770 amino acids. The tree was build using FastTree with default parameters.


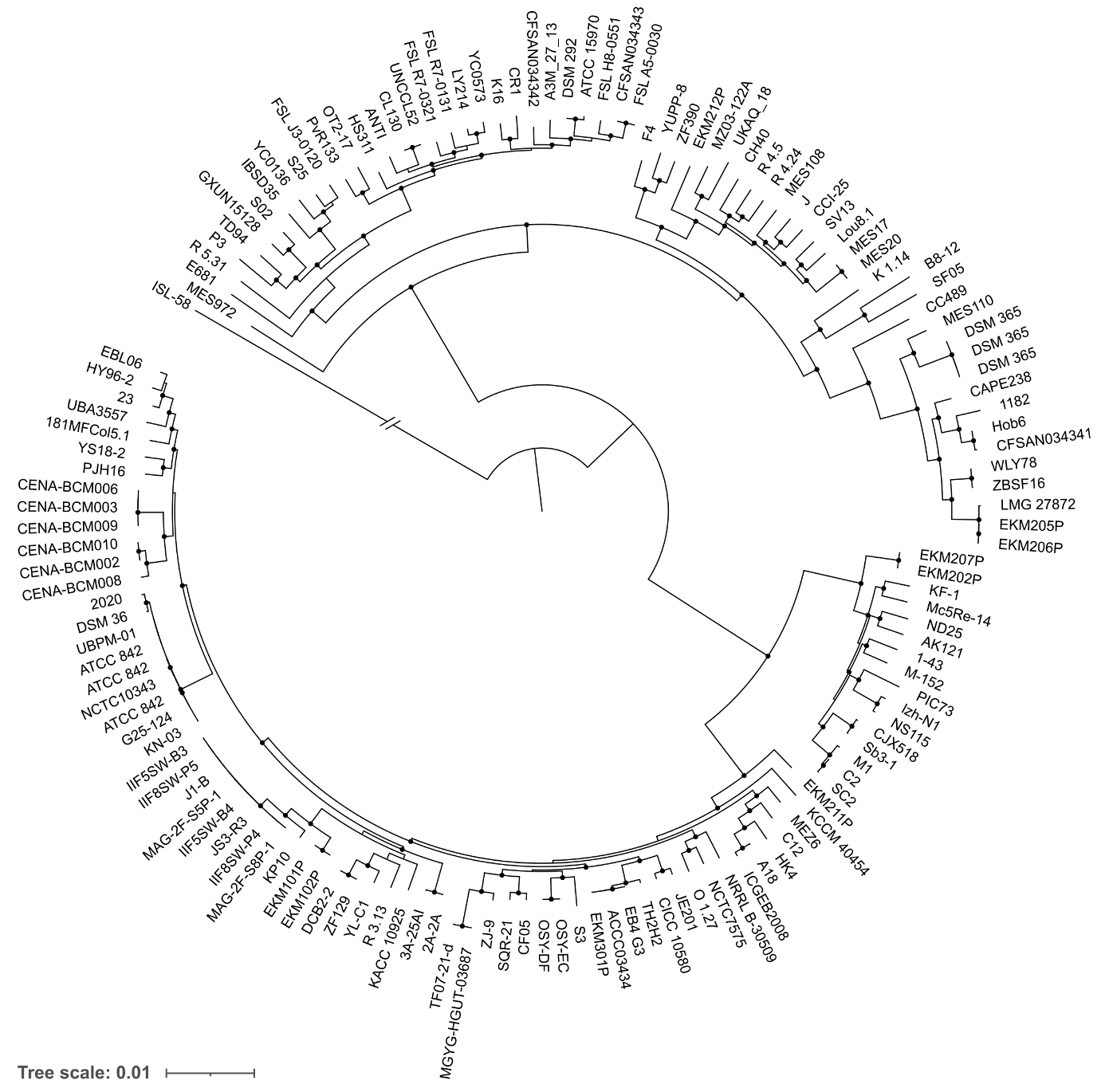


**Figure S3 Phylogenetic reconstruction of P. polymyxa affiliated strains**. Single copy orthologous protein sequences were detected and aligned using Orthofinder resulting in an alignment of 1,397 sequences for a total of 406,116 amino acids. Black dots represent local support values >95% obtained through the Shimodaira-Hasegawa test. Branch length for ISL-58 is 0.43
